# Effects of Multiple Stressors on the Spatial Pattern of Fish Diversity in the Middle and Lower Reaches of the Han River, China

**DOI:** 10.3390/ani15213109

**Published:** 2025-10-26

**Authors:** Zhiyuan Qi, Fei Xiong, Xingkun Hu, Dongdong Zhai, Le Hu, Yanfu Que, Xinbin Duan, Yuanyuan Chen, Hongyan Liu, Bin Zhu

**Affiliations:** 1 Hubei Engineering Research Center for Protection and Utilization of Special Biological Resources in the Hanjiang River Basin, College of Life Sciences, Jianghan University, Wuhan 430056, China; q15055974063@163.com (Z.Q.); zhaidongdong@jhun.edu.cn (D.Z.); hule15872813074@163.com (L.H.); yychen@jhun.edu.cn (Y.C.); lhy9603@126.com (H.L.); 2 Institute of Hydroecology, Ministry of Water Resources and Chinese Academy of Sciences, Wuhan 430079, China; huxingkun@mail.ihe.ac.cn (X.H.); yfque@mail.ihe.ac.cn (Y.Q.); 3 National Agricultural Science Observing and Experimental Station of Chongqing, Yangtze River Fisheries Research Institute, Chinese Academy of Fishery Science, Wuhan 430223, China; duan@yfi.ac.cn

**Keywords:** human stressors, natural factors, α-diversity, β-diversity, structural equation modeling

## Abstract

Human activities have altered rivers worldwide, but how their combined effects shape fish assemblages remains unclear. We therefore surveyed fish and habitats seasonally along the middle and lower reaches of the Han River, China, during 2022, specifically in June–August (wet season) and October–November (dry season). This study analyzed the spatial distribution pattern of fish diversity, explored the effects of natural factors (e.g., hydrology, water quality) and human stressors (e.g., dam, land use) on the spatial pattern of fish diversity, and identified the key driving factors. Cluster analysis and Non-metric Multidimensional Scaling (NMDS) showed that the fish communities could be divided into three groups: the Danjiangkou reservoir area (DRA), the middle reaches (MR), and the lower reaches (LR). For α-diversity, the LR had the highest value, followed by the DRA, with the MR being the lowest. For β-diversity, the MR had the highest value, followed by the LR, with the DRA being the lowest. Random Forest model showed that fish diversity was mainly affected by natural factors; among these factors, the key drivers of α-diversity were hydrological factors such as the water level (3.56–5.97%) and river width (4.53–4.69%), while for β-diversity, the key drivers were water quality factors, including the dissolved oxygen (10.08–12.36%), total nitrogen (6.49–9.31%), and chlorophyll a (8.26–8.40%). Structural Equation Modeling further revealed that natural factors affected β-diversity mainly through direct pathways, while human stressors affected β-diversity mainly through indirect pathways. The results revealed the differential roles of natural factors and human stressors in driving the patterns of fish α-diversity and β-diversity in human-disturbed rivers, which will provide a scientific basis for the conservation of fish diversity in the Han River.

## Simple Summary

Understanding the relationship between the environment and biodiversity is crucial for biodiversity conservation. However, fish diversity in rivers is affected by many factors, such as the natural environment and human activities. Therefore, we selected the middle and lower reaches of the Han River in China—an area significantly impacted by human activities—as the research region to identify the main factors affecting fish diversity and how these factors worked.

## 1. Introduction

Understanding the relationship between environmental conditions and biodiversity is a central theme in ecological research. Elucidating the mechanisms through which environmental drivers shape biodiversity patterns is crucial for mitigating the global biodiversity crisis and ensuring ecosystem sustainability [1,2]. Since the beginning of the 21st century, escalating anthropogenic pressures have posed unprecedented threats to global biodiversity [3,4]. Among various ecosystems, freshwater ecosystems have been impacted particularly severely, with river fish diversity increasingly affected by multiple stressors from various human activities: multiple stressors such as interrupted river connectivity, altered hydrological regimes, and aggravated water pollution caused by dam construction are systematically damaging the ecological integrity of river ecosystems [5,6,7].

In freshwater ecosystems, natural environments (e.g., hydrology, water quality) are fundamental components of habitats, closely associated with fish distribution and influencing the patterns of fish diversity. Habitat modification directly shaped the spatial structure of fish communities [6,8]. Hydrological factors such as water level and water temperature affected fish distribution by altering habitat structure [8,9,10], while water quality parameters including nutrients and dissolved oxygen influenced fish survival through environmental filtering [11]. Additionally, the impacts of human activity stressors such as dam construction and land use on fish diversity have become increasingly prominent [8,9]. Guo et al. demonstrated that these human pressures had already left detectable fingerprints on freshwater fish biodiversity worldwide [12].

The formation of fish diversity patterns is synergistically driven by multiple drivers [13]. However, traditional studies often analyzed single types of environmental factors, such as hydrology [14], geomorphology [15] or land use [16], in isolation, thereby overlooking their interactions. This limitation hampers a full understanding of the direct and indirect pathways by which environmental variables shape diversity [17]. Against this background, investigating how natural factors and human stressors jointly affect the spatial patterns of fish diversity in rivers under intense human disturbance has become an urgent need for formulating effective conservation strategies [18,19], and identifying the key driving factors of fish diversity under multiple stressors is a common concern of scientific researchers and managers.

The Han River, the largest tributary of the Yangtze River, originates in Shaanxi Province and flows 1570 km southeast through Shaanxi and Hubei Provinces before joining the Yangtze in Wuhan [20,21]. The elevation drops from ~1700 m in the headwaters to ~20 m at the confluence. Since the completion of the Danjiangkou Reservoir, seven cascade dams have been commissioned in the middle and lower reaches of the Han River [22]. Regulated hypolimnetic releases and peaking operations have altered thermal and flow regimes, disrupting spawning cues and reducing recruitment [16]. Urbanisation of the middle basin has converted riparian vegetation to impervious surfaces, increasing surface runoff and nutrient loading that degrade water quality [23,24]. Channel encroachment has further contracted available habitats. Although fish communities of the upper Han were relatively well documented, ecological data for the heavily modified middle–lower reaches remain scarce [16,25,26]. Quantifying the dominant drivers of fish diversity in this multiple-stress context is therefore a prerequisite for evidence-based conservation.

During 2022 we conducted seasonal surveys of fish assemblages and their habitats in the middle–lower Han River. We quantified spatial patterns of taxonomic and functional α-diversity and β-diversity, disentangled the relative influences of natural drivers (hydrology, water quality) and anthropogenic stressors (dam density, land use change), and identified the key predictors for each diversity component. Specifically, the aims were to clarify the following questions: (1) How did α-diversity and β-diversity of fish vary spatially under multiple stressors? (2) Which natural or human stressors dominated α-diversity versus β-diversity? (3) Did stressors affect β-diversity directly or indirectly?

## 2. Materials and Methods

### 2.1. Study Area and Sampling Site Setup

The physical geography of the middle and lower reaches of the Han River covers the section from Danjiangkou to Hankou, with a total length of approximately 652 km. The river channel morphology is distinctly differentiated: the reach within the Danjiangkou Reservoir maintains a constant water level of 160 m year-round, forming a stable lentic habitat in the reservoir area with prolonged water retention time; the middle reach, spanning from Laohekou city to Zhongxiang city, features a hilly valley landscape, where 5 cascade dams have been built, leading to severe habitat fragmentation and the division of the originally continuous river channel into multiple isolated reservoir sections; the lower reach enters the meandering channel of the Hanjiang Plain. Affected by the water diversion of the South-to-North Water Diversion Project, the water surface width varies, while the overall connectivity of the river remains relatively high.

The spatial pattern of land use in the middle and lower reaches basin of the Han River shows obvious differentiation. In the upstream area of the basin (above Danjiangkou), the land is mainly covered by natural vegetation, with forest ecosystems dominating, followed by water bodies and wetland systems. The overall ecosystem is less disturbed by human activities and maintains high naturalness and connectivity. In the middle reaches, urbanization has advanced significantly, and the land use pattern is characterized by the coexistence of cultivated land and highly artificial surfaces (e.g., urban areas, transportation construction land), with prominent natural habitat fragmentation. In the lower reach segments, cultivated land has become the dominant land use type.

**Figure 1:**
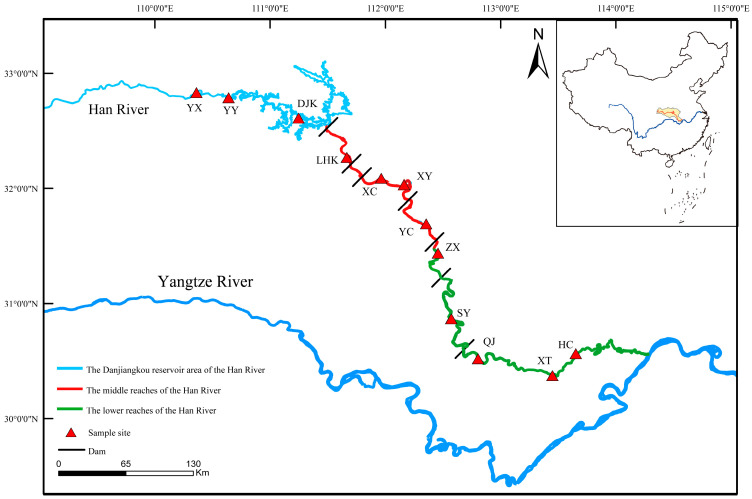
Distribution of sampling sites in the middle and lower reaches of the Han River.

Considering habitat characteristics and the impacts of human activities (e.g., dam, land use), a total of 12 survey sites were set up in the middle and lower reaches of the Han River. From above to below, these sites were: Yunxi (YX), Yunyang (YY), Danjiangkou (DJK), Laohekou (LHK), Xiangcheng (XC), Xiangyang (XY), Yicheng (YC), Zhongxiang (ZX), Shayang (SY), Qianjiang (QJ), Xiantao (XT), and Hanchuan (HC). Among them, YX, YY and DJK are located in the Danjiangkou Reservoir area; LHK, XC, XY and YC are in the middle reaches of the Han River; and ZX, SY, QJ, XT and HC are in the lower reaches (Figure 1).

### 2.2. Field Survey and Laboratory Analysis

#### 2.2.1. Fish Sampling

Two surveys of fish communities and environmental factors in the middle and lower reaches of the Han River were conducted in June–August 2022 (wet season) and October–November 2022 (dry season). To ensure data comparability, a standardized sampling method was adopted at each survey site, using two types of gear: customized gillnets and cage nets. The customized gillnets are divided into floating gillnets and sinking gillnets, which are used to collect pelagic fish and demersal fish, respectively, while cage nets primarily target benthic fish. The customized gillnets are constructed by splicing four panels of nets with different mesh sizes, including 2.0 cm, 6.0 cm, 10.0 cm, and 14.0 cm. Each net panel measures 50 m in length and 2 m in height, resulting in a total length of 200 m for the complete set of customized gillnets. The cage nets have dimensions of 18 m in length, 0.33 m in height, and 0.45 m in width, with a mesh size of 0.8 cm. One set of floating gillnets, one set of sinking gillnets, and two cage nets were used each day. The customized gillnets were deployed around 17:00–18:00 (afternoon) and retrieved around 6:00–7:00 the following morning, with a deployment duration of 12 h. The cage nets were deployed at 6:00–7:00 (morning) and retrieved around 6:00–7:00 the next morning, with a deployment duration of 24 h. For each sampling event, continuous sampling was conducted at each site for 10 consecutive days.

After retrieving the nets, fish were identified, counted, and weighed while fresh. Body length was measured to the nearest 1 mm, and body weight to the nearest 0.1 g. Live fish individuals were released back to the original site post-measurement, while dead ones were disposed of by burial. Fish species identification referred to the FishBase database to avoid issues of invalid species, synonyms, and duplicate naming (Table S1).

#### 2.2.2. Survey of Environmental Factors

In this study, 15 factors were investigated, which were categorized into natural factors (including hydrology and water quality) and human stress factors (including dam and land use) (Table S2). Water temperature (WT) and dissolved oxygen (DO) were measured on-site using a portable instrument (YSI 6600, manufactured by YSI Incorporated, located in Yellow Springs, OH, United States). Total nitrogen (TN), total phosphorus (TP), chlorophyll a (Chl-a), particulate organic matter (POM), and chemical oxygen demand (COD) were determined in the laboratory based on the Water and Wastewater Monitoring and Analysis Method [24]. For the dam factor, the number of dams built between each survey site and the downstream estuary was used as a quantitative indicator. Data on dam construction (Dam) and elevation (Elve) were obtained using the Global Positioning System (GPS) and Google Earth software (version 7.3.6). River width (RW) was calculated by extracting river boundary information using the Hydrological Analysis toolset in ArcMap 10.8, based on the high-precision Digital Elevation Model (DEM) of 2022. Land use data were derived from 30 m resolution Landsat 8 remote sensing images of GlobalLand30 (2022). Using ArcMap 10.8 software, with each sampling site as the center, remote sensing image data of land use within a 10 km buffer zone around the site were processed to obtain the coverage rate of each land use type. The land use types were classified into cultivated land (CL), forest land (FL), grassland (GL), wetland, water body (WB), artificial surface (AS), and bare land [16,27]. Water level (WL) was retrieved from the Han River Real-time Hydrological and Water Level Monitoring Website (http://szzdjc.cnemc.cn:8070/GJZ/Business/Publish/Main.html/), accessed on 30 November 2022.

### 2.3. Data Analysis

#### 2.3.1. Fish Community

Based on fish species presence-absence data (0/1), a Bray–Curtis dissimilarity matrix was constructed for cluster analysis (CLUSTER), which divided the fish communities into three groups corresponding to the Danjiangkou Reservoir area, the middle reaches, and the lower reaches. Analysis of Similarities (ANOSIM) was used to test the consistency between this cluster-based division and the artificial river basin division: if R > 0.5 and *p* < 0.01, it indicates that the community cluster division is highly consistent with the artificial river basin division, and the inter-group differences in communities are significantly greater than the intra-group differences. Meanwhile, non-metric multidimensional scaling (NMDS) was used to analyze the similarity of fish communities between different groups. The cluster analysis was conducted using the “cluster” package in R (v4.4.1), and the NMDS was performed using the “vegan” package in R (v4.4.1) [28].

#### 2.3.2. Fish Diversity Indices

Species diversity was characterized using the Shannon diversity index (*H′*) [29], Simpson dominance index (*D*) [30], and Pielou evenness index (*J′*) [31].

(1) $$H^{\prime }=-\sum_{i=1}^{s}P_{i}lnP_{i}$$

(2) $$D=1-\sum_{i=1}^{S}{\left(\frac{N_{i}}{N}\right)}^{2}$$

(3) $$J^{\prime }=\frac{H^{\prime }}{\ln s}$$

In the formulas, *S* represents the number of species, *P_i_* is the proportion of the biomass of the *i*-th species to the total biomass, *N_i_* denotes the number of individuals of the *i*-th species, and *N* is the total number of individuals of all species.

According to previous studies [32], for functional diversity, nine indicators were selected as key functional traits from three dimensions (feeding, locomotion, and reproduction), including fish trophic level, mouth position, feeding guild, maximum body length, ecological guild, body shape, average fecundity, age at first maturity, and spawning type (Table S2). Functional diversity was measured using the Functional Richness Index (FRic), Functional Evenness Index (FEve), and Functional Divergence Index (FDiv) [33,34].

(4) $$FRic=\frac{SF_{iC}}{Rc}$$

(5) $$FEve=\frac{1}{S}\sum_{i=1}^{S}\frac{W_{i}}{\sum_{j=1}^{S}W_{j}}$$

(6) $$FDiv=\frac{\sum_{i=1}^{S}\sum_{k=1}^{T}\left|X_{ik}-g_{k}\right|}{S\cdot T}$$

In the formulas, *SF_ic_* represents the niche space occupied by species *i*, *Rc* denotes the niche space occupied by functional traits in the entire community, *W_i_* is the relative abundance of species *i*, *W_j_* is the relative abundance of species *j*, *X_ik_* is the value of trait k for species *i*, *gk* is the centroid of trait *k*, *S* is the number of species, and *T* is the number of traits.

For β-diversity, the Sørensen dissimilarity index and its turnover and nestedness components were used to quantify species β-diversity [35]. To quantify functional β-diversity, the method proposed by Villéger was adopted [36], which extends Baselga’s framework to functional diversity, and the Sørensen dissimilarity (FDsor) was used to calculate functional β-diversity. Relevant calculations were performed using the “vegan” package, “FD” package, and “betapart” package in R.

(7) $$Sorensen=1-\frac{2C}{A+B}$$

(8) $$FDsor=1-\frac{2\sum_{i=1}^{S}\min\left(f_{iA},f_{iB}\right)}{\sum_{i=1}^{S}\left(f_{iA}+f_{iB}\right)}$$

In the formulas, *C* represents the number of shared species between the two communities; *A* and *B* denote the total number of species in each community, respectively; and *f_iA_* and *f_iB_* stand for the functional values of species *i* in communities *A* and *B*, respectively.

In this study, the Kruskal–Wallis H test was used to test the spatial distribution differences in α-diversity (Shannon, Simpson, Pielou, FRic, FEve, and FDiv) and β-diversity (SDsor, SDsim, SDsne, FDsor, FDsim, and FDsne) between the Danjiangkou Reservoir area, the middle reaches, and the lower reaches [26].

#### 2.3.3. Drivers of Fish Diversity

Random Forest (RF) model was used to analyze the relationship between α/β-diversity and environmental factors, and to identify the main stressor factors affecting α-diversity indices. The RF model adopted leave-one-out cross-validation, and the %IncMSE (mean decrease accuracy) index was used to estimate the relative importance of environmental factors in predicting diversity changes, thereby quantifying the environmental factors that influence diversity. The RF model was implemented using the “randomForest” package in R (v4.4.1) [26].

Structural Equation Modeling (SEM) was employed to further evaluate how environmental factors act on β-diversity. The model goodness-of-fit was tested using Fisher’s C statistic (*p* > 0.05), and the model was optimized by adding or removing paths to assess path significance. Model fit was evaluated using the corrected Akaike Information Criterion (AIC), with lower values indicating better fit to the data. R^2^ was used to represent the proportion of variance explained by the model, ranging from 0 to 1. “Effect size” was used to quantify the strength of path relationships between variables, including direct effects, indirect effects, and total effects. Indirect effects were quantified by the product of standardized path coefficients, and the absolute values of the overall effect sizes of multiple environmental factors were used as a general measure. The SEM was run using the “piecewiseSEM” package in R (v4.4.1) [37].

## 3. Results

### 3.1. Fish Community Composition

**Figure 2:**
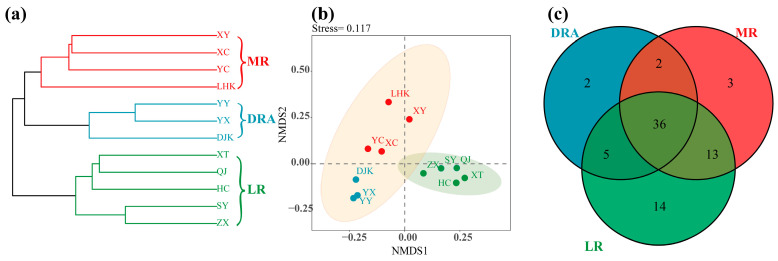
Spatial pattern and species composition of fish communities in the middle and lower reaches of the Han River. (**a**) Cluster analysis dendrogram; (**b**) NMDS ordination plot; (**c**) Venn diagram. DRA indicates the Danjiangkou Reservoir area, MR indicates the middle reaches, and LR indicates the lower reaches. DRA in blue, MR in red, LR in green. Sampling sites: YX, YY, DJK, LHK, XC, XY, YC, ZX, SY, QJ, XT, and HC.

In 2022, a total of 75 fish species were surveyed at 12 sites in the middle and lower reaches of the Han River, belonging to 7 orders and 13 families. Among these, the Cypriniformes was the most species-rich order, with 50 species (Table S1). Through hierarchical cluster analysis, the fish communities in the middle and lower reaches of the Han River were divided into three groups, corresponding to the Danjiangkou Reservoir area (DRA), the middle reaches (MR) and the lower reaches (LR) in terms of geographical space (R = 0.648, *p* < 0.001) (Figure 2a). This indicated a high consistency between the distribution of fish community structure and the geographical division.

The results of NMDS analysis showed that the fish communities in the downstream and reservoir reaches of the Han River were relatively clustered, while the fish communities at the midstream sampling sites were relatively scattered (Figure 2b). A total of 45 fish species were collected in the reservoir reach, 52 species in the midstream reach, and 66 species in the downstream reach. There were 36 fish species that occurred in all reaches (Figure 2c).

### 3.2. Spatial Patterns of α-Diversity and β-Diversity

**Figure 3:**
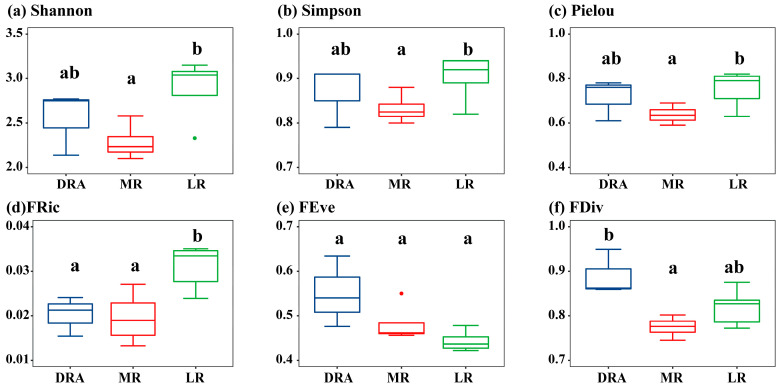
Spatial distribution of fish α-diversity in the middle and lower reaches of the Han River. (**a**) Shannon diversity index; (**b**) Simpson dominance index; (**c**) Pielou evenness index; (**d**) Functional Richness Index; (**e**) Functional Evenness Index; (**f**) Functional Divergence Index. DRA indicates the Danjiangkou Reservoir area, MR indicates the middle reaches and LR indicates the lower reaches. DRA in blue, MR in red, LR in green. In the boxplots, the letters “a” and “b” indicate significant differences between groups (*p* < 0.05).

In terms of α-diversity, the middle and lower reaches of the Han River exhibited a spatial pattern where α-diversity was the highest in the lower reaches (LR), followed by Danjiangkou Reservoir reach (DRA), and the lowest in the middle reaches (MR). For the sampling sites in each reach of the Han River, Shannon ranged from 2.10 to 3.15, Simpson from 0.80 to 0.94, and Pielou from 0.59 to 0.82 (Figure 3a–c). The Shannon (2.88 ± 0.33), Simpson (0.90 ± 0.05), and Pielou (0.75 ± 0.08) in LR were all significantly higher than those in MR (*p* < 0.05), while there were no significant differences in the overall Shannon, Simpson, or Pielou between the DRA and the MR (*p* > 0.05). Regarding the functional diversity indices of fish at the sampling sites in each reach of the Han River, FRic ranged from 0.01 to 0.04, FEve from 0.39 to 0.63, and FDiv from 0.74 to 0.95 (Figure 3d–f). The overall fish FRic in LR (0.03 ± 0.01) was the highest, which was significantly higher than those in the MR and DRA (*p* < 0.05). The overall FEve (0.55 ± 0.08) and FDiv (0.89 ± 0.05) in DRA were the highest among the three reaches, with no significant differences between different reaches (*p* > 0.05).

**Figure 4:**
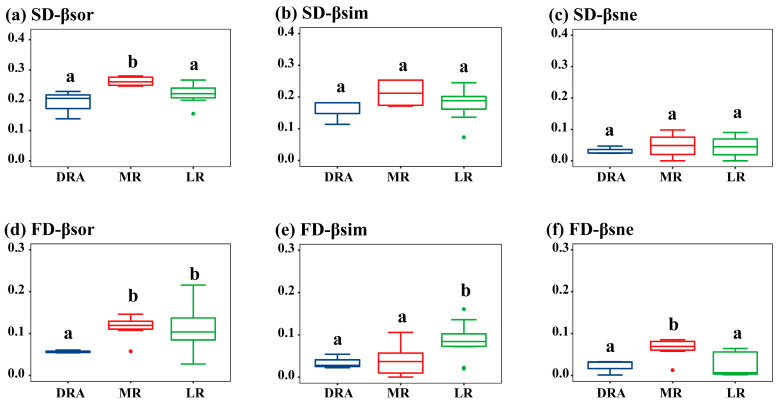
Spatial distribution of fish β-diversity in the middle and lower reaches of the Han River. (**a**) Species β-diversity; (**b**) Species β-diversity turnover component; (**c**) Species β-diversity nestedness component; (**d**) Functional β-diversity; (**e**) Functional β-diversity turnover component; (**f**) Functional β-diversity nestedness component. DRA indicates the Danjiangkou Reservoir area, MR indicates the middle reaches and LR indicates the lower reaches. DRA in blue, MR in red, LR in green. In the boxplots, the letters “a” and “b” indicate significant differences between groups (*p* < 0.05).

β-diversity exhibited a spatial pattern where it was the highest in the middle reaches (MR), followed by the lower reaches (LR), and the lowest in the Danjiangkou Reservoir reach (DRA). Among the different components of β-diversity, the turnover component dominated. For the species diversity of each reach in the Han River, the βsor values ranged from 0.14 to 0.35, βsim values from 0.07 to 0.33, and βsne values from 0.01 to 0.10 (Figure 4a–c). It could be seen that, unlike α-diversity, the βsor of fish communities in the MR was significantly higher than that in the DRA and LR (*p* < 0.05), which might be attributed to the high β species diversity caused by dam barriers between different sampling sites in the MR. There was no significant difference between the turnover and nestedness components of species β-diversity. For functional β-diversity, the βsor values ranged from 0.06 to 0.11, βsim values from 0.04 to 0.09, and βsne values from 0.02 to 0.07. In terms of functional β-diversity, both the LR and MR were significantly higher than the DRA, and the turnover and nestedness components of functional β-diversity in the DRA were the lowest (Figure 4d–f).

### 3.3. Relationships Between Fish Diversity and Environmental Factors

#### 3.3.1. Main Drivers of α-Diversity and β-Diversity

**Figure 5:**
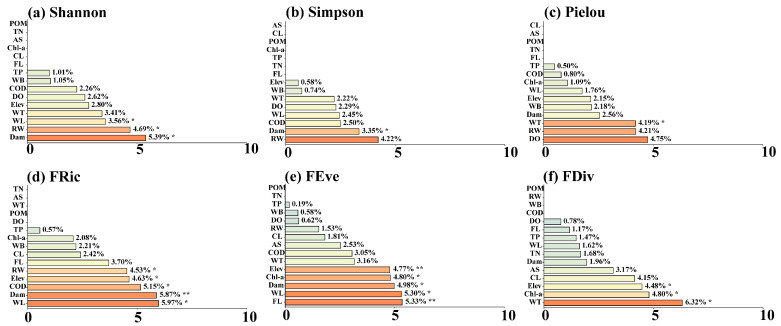
Illustrates the importance ranking plot of the effects of environmental factors on α-diversity based on the Random Forest, including (**a**) Shannon diversity index, (**b**) Simpson dominance index, (**c**) Pielou evenness index, (**d**) Functional Richness Index (FRic), (**e**) Functional Evenness Index (FEve), and (**f**) Functional Divergence Index (FDiv). Statistical significance levels are indicated by asterisks (** indicates *p* < 0.01, * indicates *p* < 0.05).

Results of the RF model showed that among the α-diversity indices, Shannon, Simpson, and FRic were mainly influenced by hydrological factors such as RW (4.53–4.69%) and WL (3.56–5.97%) (*p* < 0.05), followed by the Dam (5.39–5.87%) among human stressors (Figure 5a,d). In contrast, water quality factors and land use factors had less influence. For Pielou, FEve, and FDiv, in addition to the aforementioned environmental factors, they were also significantly correlated with FL, as well as Chl-a and DO in water quality parameters (*p* < 0.05) (Figure 5b,c,e,f). In summary, α-diversity was mainly driven by hydrological factors, followed by the dam factor, while water quality and land use had less influence.

**Figure 6:**
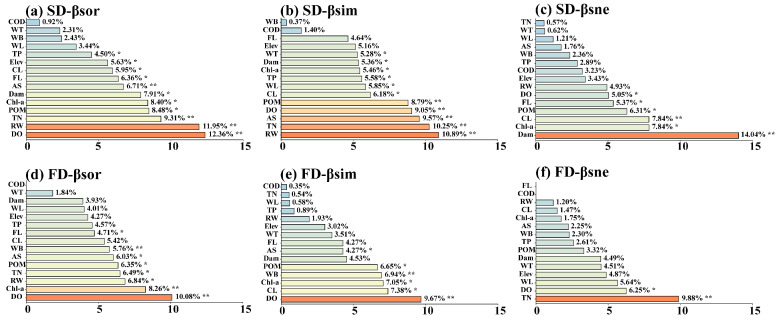
Illustrates the importance ranking plot of the effects of environmental factors on β-diversity based on the Random Forest, including (**a**) Species β-diversity (SD-βsor), (**b**) Species β-diversity turnover component (SD-βsim), (**c**) Species β-diversity nestedness component (SD-βsne), (**d**) Functional β-diversity (FD-βsor), (**e**) Functional β-diversity turnover component (FD-βsim), and (**f**) Functional β-diversity nestedness component (FD-βsne). Statistical significance levels are indicated by asterisks (** indicates *p* < 0.01, * indicates *p* < 0.05).

Regarding β-diversity, it was mainly affected by water quality factors including DO (10.08–12.36%, *p* < 0.01), TN (6.49–9.31%, *p* < 0.05), and Chl-a (8.26–8.40%, *p* < 0.05), followed by RW (6.84–11.95%, *p* < 0.05). Dam and land use had minimal influence (Figure 6a,d). Among different components of β-diversity, the contribution and significance of hydrological factors, water quality factors, dam, and land use varied. For example, land use factors such as CL, AS, and WB had significant effects on the turnover component, and Dam showed the highest contribution (14.04%) in SD-βsne (Figure 6b,c,e,f).

In conclusion, during the formation of the fish diversity pattern in the middle and lower reaches of the Han River, natural factors played a dominant role, while human stressors served as secondary driving factors. Furthermore, there were differences in the driving factors for α-diversity and β-diversity within natural factors. Specifically, α-diversity exhibited a stronger correlation with factors such as RW and WL. These factors directly defined fish habitats and community structures, determining the basic pattern of α-diversity, indicating that hydrological factors and dams dominated α-diversity. In contrast, β-diversity was more strongly correlated with water quality factors. Physicochemical parameters including DO, TN, and Chl-a affected fish survival at the micro level. By regulating local community assembly through environmental filtering, these parameters determined the differentiation of β-diversity, which suggested that water quality factors dominated β-diversity.

#### 3.3.2. Pathways of Environmental Factors Affecting β-Diversity

**Figure 7:**
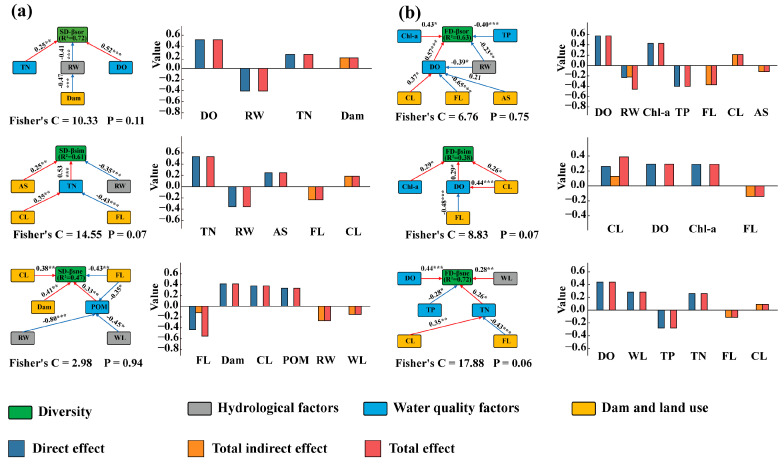
Graphical representation of the structural equation modelling for the effects of β-diversity. (**a**) Structural equation modeling for β species diversity; (**b**) Structural equation modeling for β functional diversity. The red lines indicate significant positive effects, blue lines indicate significant negative effects, and the values represent the correlation coefficient r between two variables in the paths linked by arrows. Statistical significance levels are indicated by asterisks (*** indicates *p* < 0.001, ** indicates *p* < 0.01, * indicates *p* < 0.05).

Results of the SEM showed that water quality factors and hydrological factors affected β-diversity mainly through direct effects. Among them, the direct effect values of water quality factors on β-diversity were 0.77 and 1.40, respectively, while the direct effect values of hydrological factors on β-diversity were 0.41 and 0.23, respectively. Dams and land use affected fish β-diversity indirectly by influencing water quality factors and hydrological factors, with the overall indirect effect values on β-diversity being 0.19 and 0.70, respectively (Figure 7a,b).

For the turnover and nestedness components of β-diversity, both direct and indirect effects of Dam and land use existed; they affected β-diversity mainly through indirect effects, with the overall indirect effect values exceeding the direct effect values. The indirect effect values were 0.42, 0.12, 0.26, and 0.20, respectively (the direct effect values were 0.25, 1.22, 0.26, and 0.00, respectively). In addition, the study found that the relative importance of Dam and land use in the turnover and nestedness components increased significantly, and their overall contribution exceeded that of hydrological factors and water quality factors, indicating the non-negligible importance of the indirect effects of Dam and land use.

Among the multiple stressors affecting β-diversity, water quality factors mainly had a positive impact on β-diversity. This manifested as more significant differences in the selectivity of environmental filtering, accompanied by an increase in β-diversity, which was the core mechanism by which water quality factors drove β-diversity positively. WL, Dam and CL had positive effects on β-diversity, while RW, FL, and AS had negative effects on β-diversity.

## 4. Discussions

### 4.1. Effects of Different Stressors on the Patterns of α-Diversity and β-Diversity

We observed a clear inversion of diversity patterns along the Han River: α-diversity peaked in the lower reach and was lowest in the fragmented middle reach, whereas β-diversity showed the opposite trend (highest in the middle, lowest in the reservoir). Similar reach-scale reversals have been reported worldwide [38]. Consistent with findings from the Yangtze basin [13], natural drivers explained the largest fraction of variance in both α and β-diversity, whereas anthropogenic stressors were secondary. Crucially, our results disentangled the mechanistic basis of this dominance: hydrological variables (WL, RW) governed αdiversity, whereas water-quality gradients (DO, TN, Chl-a) controlled β-diversity.

Hydrological drivers governed α-diversity by creating heterogeneous habitat templates [39]. Variation in water level and channel width generated niche axes that accommodated species with contrasting flow and depth preferences, thereby elevating local richness [38,40]. Dam construction along the middle Han has eliminated lotic stretches and replaced them with a chain of lentic impoundments. Fragmentation barriers curtailed dispersal between the reservoir and the lower reach, depressing species richness in the middle reach [41]. Conversely, the lower reach received colonists from the Yangtze species pool, boosting α-diversity [42,43,44].

Habitat homogenisation also eroded functional diversity. Impounded sections filtered for lentic-tolerant traits, increasing the proportion of functionally similar species and reducing functional richness [45,46]. As predicted by environmental filtering theory, isolated reaches with uniform hydraulics converged towards trait similarity [47,48] and hydrological alteration was the primary driver of this functional convergence in the middle Han [49,50].

β-diversity reflected spatial differences in species composition, while water quality factors more often exert micro-level influences on fish survival by regulating physicochemical parameters such as DO, TN, and Chl-a, further affecting the distribution of fish across different regions and thus emerging as important driving factors of β-diversity [51]. Changes in the concentrations of nutrients such as nitrogen and phosphorus directly affected the growth and community structure of phytoplankton, which then affected the species composition and abundance of fish at higher trophic levels through food chain transmission [52]. The uneven distribution of such resources exposed fish in different regions to different competitive pressures, further exacerbating differences in fish community composition between different river sections [53,54,55]. Additionally, the study found that human stressors such as land use and dam construction were secondary factors. The expansion of cropland and artificial surfaces in the middle reaches of the Han River has increased the discharge of nutrients such as nitrogen and phosphorus from surface and groundwater [56]. These pollutants were retained and accumulated in the slow-flowing reservoir areas formed by cascade dams, triggering water eutrophication, which collectively led to significant deterioration of water quality in this river section and affects fish β-diversity [57]. Dam construction further enhances the spatial isolation effect by forming highly fragmented habitats, restricting the movement and dispersion of fish. This, in turn, exacerbated differences in species composition between different regions, resulting in the spatial pattern where β-diversity was the highest in the middle reaches [58].

### 4.2. Direct and Indirect Effects of Different Pressure on β-Diversity

The impact of multiple stressors on fish β-diversity is a complex and multi-dimensional issue. In this study, fish β-diversity was comprehensively influenced by natural factors and human stressors, with natural factors dominating; however, there were significant differences in their modes of action. This study showed that natural factors affected β-diversity through direct action, while human stressors mainly affected β-diversity through indirect action. This phenomenon reflected the easily overlooked potential impact of human activities in shaping fish diversity.

In terms of the impact on changes in β-diversity, water quality factors affected β-diversity through direct action and were the main driving force determining overall β-diversity [59,60]. Water quality factors mainly had a positive impact on β-diversity: the higher the spatial heterogeneity of water quality, the more significant the difference in the selectivity of environmental filtering, which in turn promoted an increase in β-diversity. Changes in the concentrations of nutrients such as nitrogen and phosphorus could affect the degree of water eutrophication and the distribution of algae [53], indirectly altering the food sources and habitats of fish and leading to differences in fish distribution [61,62].

Land use change and dam placement indirectly influenced β-diversity by modifying water quality and hydrology. For both turnover and nestedness components, indirect effects calculated by our piecewise SEM were larger than direct effects, corroborating Fugère et al. [63]. Cropland expansion intensified nutrient heterogeneity between reaches, enhancing environmental filtering, and thus, promoting turnover, whereas forest regrowth homogenises water chemistry, reducing the β-diversity [55]. Dams amplified these indirect pathways by creating serial impoundments with contrasting retention times and thermal regimes. Long-residence reservoirs accumulated nutrients and warm surface waters, favouring tolerant generalists and increasing compositional differences between impounded and free-flowing sections [61]. Conversely, cold-tailwater stretches below dams lost sensitive species and became nested subsets of upstream assemblages. As physical barriers, dams also curtailed dispersal, reinforcing the indirect, environment-mediated divergence of communities [41,64].

### 4.3. Management Recommendations

This study systematically identified the key environmental factors shaping different dimensions of fish diversity in the middle and lower reaches of the Han River under multiple stressors. It also clarified the impacts of human activities, such as dam construction and changes in land use types, on fish diversity in the Han River, thereby providing a scientific basis for fish diversity conservation.

Based on the above conclusions, we propose management strategies:

(1) For dam management: It is recommended to ensure the normal operation of fish passage facilities and enhance the connectivity between river sections upstream and downstream of dams. Additionally, monitoring and evaluation of fish passage effectiveness should be strengthened, and fish passages should be optimized and improved to enhance their efficiency in facilitating fish migration. Meanwhile, scientific joint ecological scheduling of cascade hydropower projects is suggested to promote the restoration of natural hydrological processes [65] and support fish reproduction, growth, and development.

(2) For land use: The vegetation coverage along river banks should be expanded, while cropland and artificial construction land should be reduced to improve the water body’s self-purification capacity and water quality. Simultaneously, ecological buffer zones with a width of no less than 50 m should be demarcated to provide spawning substrates for fish and shelters for juvenile fish [66,67].

The main novelty of this study lied in taking the Han River, the largest tributary of the Yangtze River, as a case study to explore the impacts of multiple stressors on fish diversity in highly disturbed rivers. It revealed the differences in key driving factors and modes of action that shaped the spatial patterns of α-diversity and β-diversity, thereby enhancing the understanding of the mechanisms underlying the formation of freshwater fish diversity patterns under natural factors and human stressors. However, this study still has certain limitations: the research area is limited to the middle and lower reaches of the Han River, which may restrict the generalizability of the conclusions to a larger spatial scale. Based on this, future studies can expand the research area to the entire Han River basin. Through cross-regional comparative analysis, the general laws governing the relationship between environmental heterogeneity and fish diversity can be further verified, providing more targeted theoretical support for biodiversity conservation at different scales.

## 5. Conclusions

First, there were differences in the spatial patterns of α-diversity and β-diversity: α-diversity was the highest in the lower reaches and the lowest in the middle reaches; β-diversity was the highest in the middle reaches and the lowest in the Danjiangkou reservoir area.

Second, natural factors dominated the spatial patterns of α-diversity and β-diversity in the middle and lower reaches of the Han River, while human stressors played a secondary role. Among natural factors, hydrological factors dominated α-diversity, and water quality factors dominated β-diversity.

Third, natural factors (e.g., hydrological factors and water quality factors) mainly affected β-diversity through direct effect, while human stressors (e.g., dam and land use) affected β-diversity through indirect effect.

Finally, we suggest reconstructing the longitudinal connectivity of the river by improving the effectiveness of fish passages and regulating the ecological operation of dams. At the same time, it is recommended to increase the coverage of riparian aquatic vegetation to provide suitable habitats for fish.

## Data Availability

Data are contained within the article and Supplementary Materials.

## Supplementary Materials

The following supporting information can be downloaded from https://www.mdpi.com/article/10.3390/ani15213109/s1, Table S1: List of fish species and their functional traits in the middle and lower reaches of the Han River, China.; Table S2: Environmental factors in the middle and lower reaches of the Han River, China.
