# Supplementary material for: Effects of Multiple Stressors on the Spatial Pattern of Fish Diversity in the Middle and Lower Reaches of the Han River, China
Source: Animals (Basel). 2025 Oct 26;15(21):3109. doi: 10.3390/ani15213109 (PMC12610983; doi:10.3390/ani15213109)
Supplement: Supplementary file 1 [file animals-15-03109-s001.zip › animals-3915349-supplementary.pdf]

Supplementary Materials

Table S1 List of fish species and their functional traits in the middle and lower reaches of the Han River, China.

**Species**	**Code**	**Trophic level**	**Water layer**	**Feeding taxa**	**Maximum body length (cm)**	**Ecological type**	**Body size**	**Average absolute fecundity (grain)**	**age at sexual maturity (year)**	**spawning type**
Leiocassis albomarginatus	BBNC	3.7	D	PI	20.8	R	F	1367	2	AD
Siniperca scherzeri	BG	3.9	D	PI	36.8	E	CO	46829	2	DR
Abbottina rivularis	BHY	3.3	D	O	13.6	E	CO	1319	1	DE
Hemiculter bleekeri	BSC	2.1	P	O	18.5	L	CO	5877	1	DR
Parabramis pekinensis	BIAN	2.0	P	H	35.9	L	CO	86400	2	PE
Hemiculter leucisculus	CAN	2.8	P	O	13.4	L	CO	4216	1	AD
Ctenopharyngodon idella	CY	2.0	D	H	91	E	CY	656053	4	DR
Squaliobarbus curriculus	CYZ	2.7	M	O	43	E	CO	200264	2	DE
Hemibarbus labeo	CH	3.4	D	O	42	R	CY	31900	2	AD
Mastacembelus aculeatus	CQ	3.3	D	PI	13.5	R	CO	5000	1	PE
Leiocassis crassilabris	CCW	3.4	D	PI	28	R	F	1488	1	AD
Chanodichthys dabryi	DSB	3.3	M	PI	32.7	L	F	15366	2	AD
Hemibagrus macropterus	DQH	3.6	D	PI	29	R	F	763	2	AD
Acheilognathus macropterus	DQY	2.0	D	PI	9	L	CY	1343	1	LM
Siniperca kneri	DYG	3.9	D	PI	36.8	E	CO	46829	2	DR
Protosalanx hyalocranius	DYY	3.0	D	Z	21	L	CY	3000	1	DR
Coilia brachygnathus	DHJ	3.5	P	PI	32.5	L	CO	9999	1	DE
Elopichthys bambusa	GAN	2.5	P	PI	129.5	E	CO	588	4	DR
Acheilognathus hypselonotus	GLY	2.1	D	O	8	L	CO	1183	1	DR
Ochetobius elongatus	GUAN	3.4	M	PI	33.7	R	CY	19300	4	PE
Saurogobio gymnocheilus	GCSJ	3.3	D	O	18	R	CY	14690	1	DR
Pelteobagrus nitidus	GZHSY	3.6	D	PI	14.2	E	F	2916	1	AD
Siniperca chuatsi	GUI	4.5	D	PI	51.6	E	CO	118190	2	DR
Sarcocheilichthys nigripinnis	HQQ	3.3	D	O	110	E	CO	446	1	PE
Culter erythropterus	HQYB	4.4	D	PI	30	L	CO	19752	2	AD
Parabotia fasciata	HBFSQ	3.3	D	O	10.5	R	CO	17602	2	DR
Hemibarbus maculatus	HH	3.5	D	PI	30	E	CY	24501	2	AD
Sarcocheilichthys sinensis	HQ	3.3	D	O	173	R	CO	3310	1	PE
Tachysurus fulvidraco	HSY	3.5	D	PI	19	E	F	1173	1	AD

Xenocypris davidi	HWG	2.6	D	O	45	L	CO	101038	2	AD
Carassius auratus	JI	2.0	D	O	31.5	E	CO	3460	1	AD
Hyporamphus intermedius	JXZ	4.0	P	Z	14.1	E	CY	13000	1	DE
Cyprinus carpio	LI	3.1	D	O	90	E	CO	543652	2	AD
Hypophthalmichthys molitrix	LIAN	2.0	P	Z	92	E	CO	302219	3	DR
Opsariichthys bidens	MKY	3.3	P	PI	17.8	R	CO	2527	1	DR
Cirrhinus mrigala	MRJLL	2.2	D	Z	24.5	R	CY	550000	2	PE
Pseudorasbora parva	MSY	3.1	D	O	11.3	E	F	462	1	AD
Culter mongolicus	MGB	3.4	P	PI	44.9	L	CO	161518	2	AD
Silurus meridionalis	NFN	4.2	D	PI	39.3	E	CO	38571	4	AD
Misqurnus anquillicaudatus	NQ	3.2	D	O	20.2	E	CY	5336	1	AD
Culter oxycephaloides	NJTB	3.3	P	PI	60.2	E	CO	41517	2	DR
Silurus asotus	NIAN	4.4	D	PI	52.3	E	CO	15503	1	AD
Pseudolaubuca sinensis	PY	3.2	P	O	17	E	CO	3400	2	DR
Culter alburnus	QZB	3.4	P	PI	57.3	L	CO	306793	2	DR
Pseudobagrus truncatus	QWNC	3.4	D	O	20.8	R	F	1488	2	AD
Ctenopharyngodon idellus	QY	3.2	D	PI	117	E	CY	681119	5	DR
Megalobrama terminalis	SJF	3.3	M	O	37.7	E	CO	57000	2	PE
Cyprinus carpio specularis	SLJL	3.1	D	O	24.94	E	F	344675	3	AD
Odontobutis obscurus	STL	3.2	D	PI	17.8	L	F	491	1	AD
Saurogobio dabryi	SJ	3.3	D	O	17.6	E	CY	3179	1	DR
Pseudobrama simoni	SB	2.7	D	O	18.6	E	CO	6681	2	DR
Sinibrama taeniatus	SCHB	3.2	M	O	12	R	CO	2734	1	AD
Neosalanx taihuensis	THXYY	5.0	D	Z	8	L	CY	3000	1	DE
Coreius heterodon	TY	3.4	D	O	33.5	R	CY	20308	3	DR
Megalobrama amblycephala	TTF	3.4	D	H	31.5	E	CO	78000	2	AD
Pelteobagrus vachelli	WSHSY	3.5	D	PI	24.8	E	F	4948	2	AD
Rhinogobio typus	WJ	3.4	D	O	44	R	CY	29460	1	AD
Channa argus	WL	4.4	D	PI	52.2	E	CY	15427	2	PE
Xenocypris microlepis	XLG	2.6	D	O	22.4	L	CO	78629	2	AD
Pseudobagrus pratti	XTLC	3.4	D	O	16.2	E	F	3955	1	AD
Micropercops swinhonis	XHYY	3.2	D	PI	10.8	E	F	2462	1	AD
Acheilognathus chankaensis	XKY	2.1	D	O	8	L	CO	1183	1	DR
Xenocypris argentea	YG	2.6	D	O	23.5	E	CO	30951	2	DR
Squalidus argentatus	YJ	3.3	D	O	10.8	E	CY	6121	2	DR
Aristichthys nobilis	YONG	2.8	P	Z	120	E	CO	368816	4	DR
Pseudobagrus tenuis	YWNC	3.7	D	O	17	L	F	2684	3	AD
Distoechodon tumirostris	YWG	2.6	D	O	26.1	L	CO	137862	2	AD
hybrid sturgeon	ZKX	4.4	D	PI	85.6	R	F	1052000	2	DE

Pelteobagrus eupogon	CXHSY	3.4	D	PI	24.2	E	F	3012	2	AD
Cobitis sinensis	ZHHQ	3.3	D	O	9.4	E	CO	2559	1	DE
Rhodeus sinensis	ZHPP	2.7	D	O	4.7	E	CO	156	1	LM
Rhinogobius giurinus	ZLWXH	3.2	D	PI	10.8	E	F	2462	1	AD
Leptobotia taeniaps	ZBQ	3.3	D	PI	12.4	R	CO	1501	2	DR

Note: P: Pelagic; M: Meso-pelagic; D: Demersal. H: Herbivorous; PI: Piscivore. O: Omnivorous; Z: Zooplankton. R: Rheophilic; E: Eurytopic; L: Limnophilic. F: Fusiform; CO: Compressiform; CY: Cylindrical. AD: Adhesive eggs; DE: Demersal eggs; DR: Drifting egg; PE: Pelagic eggs.

Table S2 Environmental factors in the middle and lower reaches of the Han River, China.

Type	Group	Factors	Code	RA (Mean ±SD)	Mid (Mean ±SD)	Down (Mean ±SD)
Natural factors	Water quality	Total nitrogen (mg/L)	TN	2.59±0.21	3.49±0.98	2.24±0.72
		Total phosphorus (mg/L)	TP	0.04±0.01	0.06±0.04	0.11±0.01
		Chlorophyll a (ug/L)	Chl.a	1.5±0.44	3.97±1.63	13.53±5.73
		Particulate organic matter (mg/L)	POM	8.89±6.15	10.84±2.99	8.87±6.03
		Permanganate Index (mg/L)	COD	12.85±8.84	17.99±6.79	47.53±30.32
		Dissolved oxygen (mg/L)	DO	7.17±0.2	7.25±1.12	6.88±0.67
	Hydrology	River Wide (m)	RW	679.89±390.53	967±235.77	418.07±208.86
		Water level (m)	WL	173.64±9.24	97.68±43.65	30.17±7.83
		Water temperature (°C)	WT	31.83±0.35	22.53±3.95	29.68±0.41
	Elevation	Elevation (m)	Elev	150.46±7.69	40.03±10.55	13.48±6.14
Human stressors	Land use	Percentage of Cropland (%)	CL	0.25±0.08	0.56±0.17	0.73±0.08
		Percentage of Forest (%)	FL	0.49±0.24	0.09±0.09	0.01±0.01
		Percentage of Grassland (%)	GL	0.1±0.05	0.02±0.03	0.01±0.01
		Percentage of Water body (%)	WB	0.14±0.12	0.08±0.03	0.04±0.02
		Percentage of Artificial surface (%)	AS	0.01±0.01	0.22±0.16	0.19±0.09
	Dam	Dam	Dam	7±0	4.25±1.26	0.6±0.89
